# Interaction Between Transcranial Random Noise Stimulation and Observation-Execution Matching Activity Promotes Motor Cortex Excitability

**DOI:** 10.3389/fnins.2019.00069

**Published:** 2019-02-07

**Authors:** Fengxue Qi, Michael A. Nitsche, Volker R. Zschorlich

**Affiliations:** ^1^Department of Movement Science, Faculty of Philosophy, University of Rostock, Rostock, Germany; ^2^Department of Psychology and Neurosciences, Leibniz Research Centre for Working Environment and Human Factors, Dortmund, Germany; ^3^Department of Neurology, University Medical Hospital Bergmannsheil, Bochum, Germany; ^4^Faculty of Medicine, University of Rostock, Rostock, Germany; ^5^Department Ageing of Individuals and Society, Faculty of Interdisciplinary Research, University of Rostock, Rostock, Germany

**Keywords:** action observation, action execution, motor cortex excitability, mirror neurons, transcranial random noise stimulation

## Abstract

Pathways of the human mirror neuron system are activated during both, action observation and action execution, including lateralized activation of respective areas, as shown by observed right-or left-hand actions. Here, we investigated whether execution-dependent motor cortex excitability is affected by prior interaction between transcranial random noise stimulation (tRNS) and action observation. Sham or real tRNS (1 mA) was applied for 10-min over the left primary motor cortex during action observation. In the main experiments, participants received sham or real tRNS while they watched a video showing repeated tapping tasks, involving either the right-hand (Experiment 1, congruent action observation), or a mirror-reversed video showing the same performance (Experiment 2), followed by action execution of the right-hand. In control Experiments 1–3, participants received real tRNS while observing a perceptual sequence, watching a landscape picture, or observing the left-hand performing the action (the sequence was identical to Experiment 1), followed by action execution of the right-hand. In control Experiment 4, participants received real tRNS during congruent action observation, and then took 6-min rest. Motor-evoked potentials (MEP) were recorded before action observation, a perceptual sequence or a landscape picture, immediately after, and after action execution, or an interval of 6-min, dependent on the respective experimental condition. MEPs in the right first dorsal interosseous muscle increased significantly after real tRNS combined with congruent action observation, and after action execution compared to the sham session in Experiment 1 and control experiments. We conclude that prior interaction between real tRNS and action observation of mirror-matched movements modulates subsequent execution-dependent motor cortex excitability.

## Introduction

Motor cortical activity can be modified by observing others perform a matching movement known as observational learning, occurring for both explicitly and implicitly acquired motor skills (Heyes and Foster, 2002). The specific mirror activation of the movement representations included in a motor task is triggered as a result of both action observation and action execution (Koch et al., 2010). Some studies have shown observation-execution associations in the human brain. Functional neuroimaging studies provide evidence that observation or execution of an action activates a network of cortical regions, incorporating the inferior frontal gyrus, the superior and inferior parietal cortex, the primary motor cortex (M1), the premotor cortex (PMv), the dorsal PMv, and the supplementary motor area (Hari et al., 1998; Hamzei et al., 2003; Molenberghs et al., 2012; Kilner and Lemon, 2013).

The underlying mirror mechanisms result in comparable activation of motor or motor-related cortical networks when individuals are observing or conducting the identical action (Mattar and Gribble, 2005; Loporto et al., 2011). This neural system activation by observation enhances motor skill acquisition of the observer (Heyes and Foster, 2002; Nielsen and Cohen, 2008). The likely mechanism of this skill-enhancing effect of action observation might be long-term potentiation (LTP)-like plasticity of the respective regions, which is suggested to be promoted by task-related motor cortex activity and excitability enhancements (Celnik et al., 2006; Loporto et al., 2011). Action observation training is thus increasingly used for promoting motor learning processes in humans (Mattar and Gribble, 2005; Stefan et al., 2005; Loporto et al., 2011), including its application as treatment modality in motor rehabilitation (Ertelt et al., 2007; Bisio et al., 2017; Zhang et al., 2018).

Non-invasive neuromodulatory brain stimulation techniques are also increasingly probed for their capability to improve motor rehabilitation, and functional recovery, based on a similar concept, i.e., enhancement of task-related LTP-like processes, particularly in stroke (Vallence and Ridding, 2014). Transcranial electrical stimulation alters cortical excitability by a low intensity electrical current, which modulates neuronal resting membrane potentials (Nitsche and Paulus, 2000; Jamil et al., 2017). Depending on the waveform of stimulation, transcranial direct current (tDCS) stimulation is discerned from transcranial alternating current and random noise stimulation (tACS, tRNS). Prolonged application of tDCS can result in LTP-like plasticity (Nitsche and Paulus, 2001; Monte-Silva et al., 2013). Transcranial random noise stimulation (tRNS) is the application of a random electrical oscillation spectrum over the cortex in a range either between 0.1 and 640 Hz or between 101 and 640 Hz. Similar to tDCS, it has been shown to induce an increase of motor cortical excitability lasting for at least 60 min after intervention (Terney et al., 2008; Moliadze et al., 2012, 2014). Mechanistically, tRNS-induced neuroplasticity has been suggested to originate from modulation of voltage-gated sodium channels (Antal et al., 2010; Chaieb et al., 2015), and from cortical network-dependent stochastic resonance phenomena (Antal et al., 2014). Regarding functional effects, tRNS over M1 enhanced tracking skill learning (Prichard et al., 2014) and motor sequence learning task performance (Terney et al., 2008). Some studies have moreover shown that tRNS improved cognitive task performance (Cappelletti et al., 2013; Pirulli et al., 2013; Snowball et al., 2013).

In this study, we aimed to explore if the interaction between tRNS and action observation can boost motor cortex excitability alterations, and if combination of action observation with tRNS has also a boosting effect on motor performance-related excitability enhancements.

In the first experiment, we contrasted the impact of action observation (actions in which an observed finger tapping procedure is congruent with the subsequent execution of the respective hand movement) combined with real or sham tRNS on subsequent task execution. We expected that real tRNS promotes action observation-related motor cortex excitability enhancements, and that this effect subsequently strengthens execution-dependent excitability enhancements, as compared to sham tRNS.

To explore specificity of these effects, in the second experiment, we contrasted the effect of action observation (mirror-reversed relative to Experiment 1, both the hand performing the action and the sequence that was performed were incongruent with subsequent action execution) combined with real and sham tRNS on subsequent task execution. We hypothesized that real tRNS compared with sham tRNS does not facilitate observation-related and subsequent execution-related motor cortex excitability alterations.

To clarify specificity of these effects further, in the third experiment, we explored if (1) real tRNS combined with observation of a perceptual sequence, or a landscape image, followed by action execution; (2) real tRNS combined with action observation, in which the observed tapping posture is incongruent with the subsequent motor performance, and (3) real tRNS combined with action observation (identical to Experiment 1), but not followed by actively executed movements does induce similar alterations of motor cortex excitability to those obtained in Experiment 1.

## Materials and Methods

### Ethics Statement

The study was approved by the local ethics committee of the medical faculty of the University of Rostock, Germany (Identifier No. A 2016-0138) and met the standards of the *Declaration of Helsinki*. All participants gave written informed consent prior to participation in the study, and were financially compensated for their participation.

### Subjects

One hundred and thirty-three healthy adults submitted an informed consent in this study. Exclusion criteria were pregnancy, implanted medical devices, a history of epilepsy, history or presence of neurological, psychiatric or musculoskeletal disorders and other medical conditions, and left-handedness. One participant was left-handed and was excluded. Three subjects did not tolerate TMS and dropped out during the pre-test. One hundred and twenty-nine healthy adults (mean age: 24.42 ± 3.84 years; range: 18–37 years; 48 males) completed at least one experimental session. All included participants were right-handed according to the Oldfield handedness inventory (Oldfield, 1971) and had normal or corrected-to-normal vision.

Participants were randomly assigned to conduct predefined tasks, which are described below. Seventy participants performed only one task, 38 subjects participated in two tasks, and 21 participants took part in three tasks. All experimental sessions were separated by at least 1 week to prevent carryover effects.

### Transcranial Magnetic Stimulation (TMS)

Single-pulse TMS was generated by a MagPro R100 magnetic stimulator (Medtronic, Skovlunde, Denmark) with a slightly angulated figure-eight coil, type D-B80. The stimulator generated a biphasic pulse with a pulse width of 280 μs. The head of the participants was comfortably positioned in a chin-forehead rest to minimize coil to head movement. The coil was positioned over the left M1, and fixed tangentially to the skull with the handle pointing backward and laterally at an angle of about 45° to the sagittal plane. The optimal position of the magnetic coil was determined for activating the right resting first dorsal interosseous (FDI) muscle. To identify the hotspot, the coil was moved in 0.5 cm steps at a moderately suprathreshold stimulation intensity to identify the coil position which consistently elicited the largest motor-evoked potentials (MEPs). The optimal spot was marked with a soft-tip pen, and the coil was held in a fixed position by a mechanical arm (Manfrotto, Feltre, Italy). The correct position of the coil was continuously checked throughout the experimental session.

### Electromyography (EMG) Recordings

Participants were seated in a comfortable chair. Their arms were relaxed and the right hand pronated. The hand and elbow joints were comfortably semi-fixed by a pillow. Surface EMG was recorded from the right FDI muscle by a pair of Ag-AgCl cup electrodes (Hellige Baby-Electrodes; GE Medical Systems, Milwaukee, WI, United States) with an electrode surface area of 3 mm^2^ in a belly-tendon montage. A ground electrode was placed over the right lateral biceps brachii muscle. GE Healthcare electrode gel (GE Medical Systems Information Technologies GmbH, Freiburg im Breisgau, Germany) was used. EMG signals were amplified by a factor of 1000. The EMG amplifier (Biovision, Wehrheim, Germany) had an input resistance of 10 GΩ, and a bandwidth of 1–1000 Hz. The EMG signal was high-pass filtered offline via a digital second order Butterworth filter and a cut-off frequency of 5 Hz (Zschorlich, 1989). A DAQ-Card 6024 with 12-bit amplitude resolution (National Instruments, Austin, TX, United States) was used to acquire all signals at a sampling rate of 10 kHz/channel. Signals were processed by the DIAdem software (National Instruments, Austin, TX, United States).

### Measurement of Motor Cortex Excitability

The intensity of TMS was identified to the nearest 1% of the maximal stimulator output (% MSO) to elicit MEPs of about 1 mV (SI1mV, peak-to-peak amplitudes) (Hasan et al., 2012) over the left motor cortex representation of the relaxed right FDI muscle. For each block, 20 MEPs were obtained with the respective pre-determined TMS intensity. The interval between TMS stimuli was 4 s with a jitter of ± 0.5 s.

### Transcranial Random Noise Stimulation

Transcranial random noise stimulation was delivered by a battery-driven electrical stimulator (BrainSTIM, EMS, Bologna, Italy) through a pair of conductive rubber electrodes (25 cm^2^) placed in a saline-soaked sponge. The anode was positioned over the left M1 representational field of the right FDI muscle, which was determined by TMS. The cathode was placed over the contralateral supraorbital region. tRNS was applied for 10 min with a current intensity of 1 mA (peak-to-peak) with a 0 mA offset. Current density was 40 μA/cm^2^. The frequency spectrum ranged from 101 to 640 Hz. At the start of stimulation, the current ramped up for 5 s until it achieved 1 mA. For stimulation termination, current was ramped down for 5 s to avoid discomfort and peripheral sensory stimulation (Groppa et al., 2010). The electrodes were fixed on the head by two horizontal and perpendicular straps. For sham stimulation, the current was turned on for 30 s and then switched off and electrodes remained on the head until the end of the stimulation session.

### Action Observation

**FIGURE 1:**
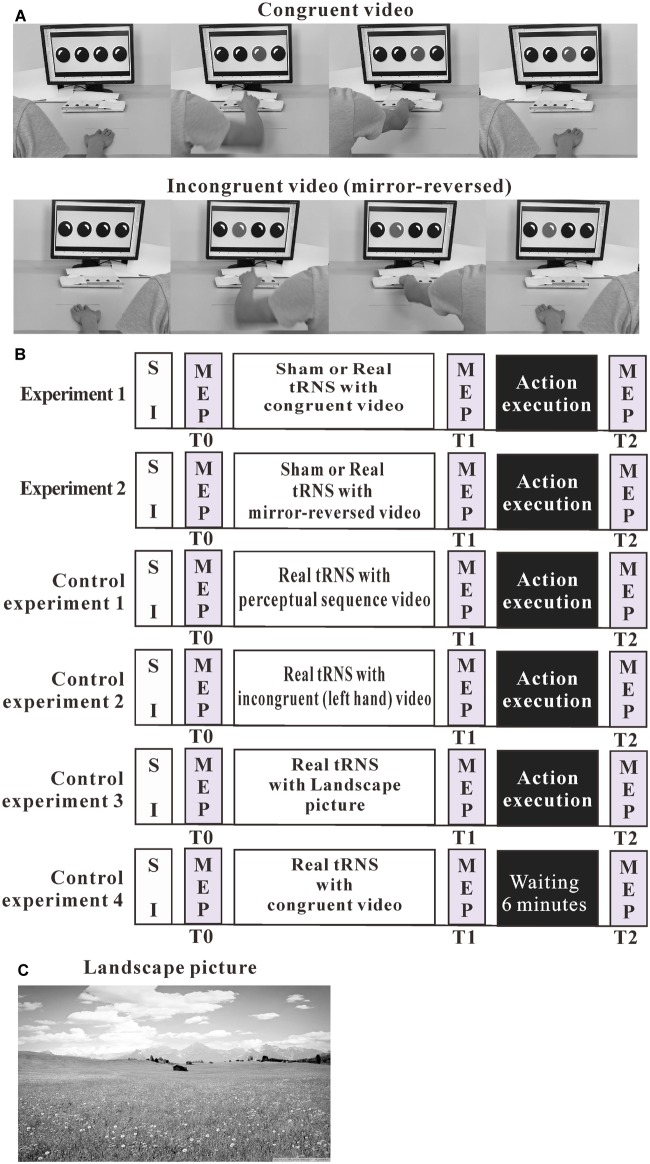
Experimental design. **(A)** Pictures are chosen from the action observation videos. The videos showed repeated goal-directed actions, including either right-hand or left-hand actions (mirror-reverse of congruent video). **(B)** The outline of the test protocols of the experiments. **(C)** Landscape picture. MEP, motor-evoked potential; SI, TMS intensity to elicit MEPs of about 1 mV amplitude.

Participants were comfortably seated in front of a 24-inch computer screen, located at 80 cm eye distance. They were instructed to maintain their hands in a relaxed position and watch one of three action observation videos. The movies included either movements of the right or the left hand. The videos displayed the respective hand resting in a pronated position on a table. A button-box was placed 20 cm in front of the hand. The four red round buttons of the box were horizontally separated by 3.5 cm (see Figure 1A).

Each video was 10 min long and included 20 short clips. These included 20 s long clips (presented 10 times) in natural speed and 40 s long clips (presented 10 times) at half of the natural speed. A 20-s long clip was always followed by a 40-s long clip and vice versa. Moriuchi et al. (2014) reported that low-speed movements have a better effect of enhancing motor cortex excitability (Moriuchi et al., 2014). The clips showed a simple index finger tapping on the round buttons in an orderly sequence. This sequence was shown 20 times in every video. If a black spot on the computer screen changed into a red spot, a human hand reached the button immediately and pressed it with the index finger only (other fingers shrank), and quickly returned to the resting position afterward. Since attention is an important mediator of neuroplasticity (Stefan et al., 2004; Kamke et al., 2012), participants were instructed to pay attention to the hand, in particular, the performing finger and the task being performed, and to count the number of a button presses. Participants verbally reported the number of button presses at the end of the 10-min video.

### Action Execution

An action execution task was performed after action observation. A custom-made button-box (4 red round buttons) and a 24-inch computer screen were placed on the table in front of the participant. Each of the red round buttons corresponded to a response key from left to right (1, 2, 3, and 4, respectively). The arrangement was identical to the action observation video (see Figure 1A).

Participants were instructed to press these buttons with the index finger of the right hand only (other fingers shrank). The distance between the button-box and the hand was 20 cm. The computer screen showed four black spots corresponding to the four buttons. If one of the four black spots changed into a red spot, the participant was instructed to reach for the button immediately, press it with the index finger only, and quickly return to the resting position afterward. One of the black spots regularly changed into a red spot every 3 s in a modeled response sequence (1, 3, 3, 1, and 4, respectively). The left hand always maintained in the resting location. The sequence was repeated eight times and the duration of the action execution task was 160 s.

### Experimental Design

We conducted three experiments. In these experiments, cortical excitability was recorded before action observation, observation of a perceptual sequence or presentation of a landscape picture, combined with sham or real tRNS (T0), immediately after action observation (T1), and after action execution or an interval of 6-min rest (T2). See Figure 1B.

#### Experiment 1: Congruent Motion Trajectories Between Action Observation and Action Execution

Twenty-six subjects (age: 24.92 ± 4.09 years; 13 females) participated in the sham tRNS session and twenty-seven participants (age: 25.67 ± 3.64 years; 17 females) took part in the real tRNS session. In this experiment, participants received sham or real tRNS during action observation, followed by action execution. Participants were expected to press the buttons in the same sequence (1, 3, 3, 1, and 4, respectively) as they had seen it executed by the right index finger in the video.

#### Experiment 2: Incongruent (Mirror-Reversed) Motion Trajectories Between Action Observation and Action Execution

Twenty-six participants (age: 24.35 ± 2.97 years; 19 females) participated in the sham tRNS session and twenty-six participants (age: 25.92 ± 2.77 years; 14 females) took part in the real tRNS session. Also in this experiment, subjects received sham or real tRNS during action observation, followed by action execution. In this video, the movement was performed with the left index finger; otherwise, the video was identical to that of Experiment 1 (mirror-reversal, using Adobe After Effects CS6). The sequence was 4, 2, 2, 4, and 1, respectively. The action execution required was identical to that in Experiment 1 (sequence 1, 3, 3, 1, and 4, respectively), and required button presses with the right hand.

#### Experiment 3: Control Experiment

In the first control experiment, we examined whether real tRNS combined with observation of a perceptual sequence, which does not require action performance, followed by action execution results in a similar cortical excitability increase as that achieved in Experiment 1. Twenty-six participants (age: 24.12 ± 3.77 years; 18 females) were recruited. Subjects watched the respective video during real tRNS over the left M1, followed by action execution (identical to Experiment 1). The video was identical to Experiment 1, but the bottom half of the screen was covered, and participants thus watched the perceptual sequence only. Participants were instructed to pay attention to the sequence, and to count the number of black to red spot changes on the computer screen. Participants verbally reported this number at the end of the 10-min video.

In the second control experiment, we investigated if observation of the left hand performing the action while receiving real tRNS followed by execution of the same action with the right hand results in similar cortical excitability alterations as compared to Experiment 1. Twenty-six subjects (age: 23.12 ± 3.88 years; 16 females) received real tRNS during action observation and then performed the respective action. In this video, the movement was performed with the left index finger and the sequence was identical to Experiment 1 (sequence 1, 3, 3, 1, and 4, respectively). The action execution required was identical to that in Experiment 1.

In the third control experiment, we explored if action execution preceded by tRNS without action observation results in the same effect on MEP as when action observation was antecedent to execution. This group watched a landscape picture during tRNS. Twenty-six participants (age: 24.85 ± 4.03 years; 16 females) were recruited. Participants watched the landscape picture during real tRNS over the left M1, followed by action execution, as described for the other experiments. The color landscape picture was derived from Google images (see Figure 1C).

In the fourth control experiment, we explored whether real tRNS combined with action observation, but without action execution, promotes similar cortical excitability alterations as those obtained in Experiment 1. Twenty-six participants (age: 23.50 ± 4.04 years; 17 females) were recruited. Subjects watched the respective video during real tRNS over the left M1. The action observation video was identical to that used in the congruent experiment, which showed the right index finger button press. The experimental conditions were identical to those in the congruent experiment, with the exception that participants did not actively execute movements. Participants waited for 6 min after action observation combined with tRNS, and the T1 MEP measure. Afterward, the T2 MEP measures followed. The six minutes break duration covered the time course of movement execution in Experiment 1.

### Statistical Analysis

Statistical analyses were performed using SPSS (version 20.0; IBM) and Prism (Version 6; GraphPad Software). A one-way analysis of variance (ANOVA) was used to examine differences for all experiments of the subjects’ age, SI1mV (%MSO) and baseline MEP amplitudes. A χ^2^ test examined gender distribution between groups.

The average peak-to-peak amplitude of 20 MEPs was calculated for each of these blocks individually. MEPs were rejected from post-processing if an EMG burst of more than 50 μV amplitude in the 300 ms time-window before TMS was identified via visual inspection of the data. These events indicated voluntary muscular activation, which has a prominent impact on MEP amplitudes (Feurra et al., 2011). In the real tRNS session of Experiment 1, one outlier was removed from the analyses because one participant’s MEP values were more than 3.52 standard deviations from the mean of all individuals (for each time point separately).

Multi-level modeling was used to analyze MEP amplitude data. For this analysis, time (T0, T1, and T2), group (eight levels: sham and real tRNS in main Experiments 1 and 2, real tRNS in the control Experiments 1, 2, 3, and 4), and interactions between time and group were included as fixed effects (include intercept). The subject-level intercept was nested into the model as random effect. The Restricted Maximum Likelihood estimation method was applied to limit any issues with underestimated variance. The Akaike’s Information Criterion (AIC) and Schwarz’s Bayesian Criterion (BIC) evaluated the fit and relative quality of the models. AIC and BIC are used to compare different candidate models and evaluate which model best fits the data. When comparing models, the smallest AIC and BIC indicate a better fitting model. Fisher’s *post hoc* tests were performed for multiple comparisons in all experiments. Effect sizes were calculated by Cohen’s *d* (Cohen, 1988) to estimate the magnitude of the difference MEP amplitude changes between groups. The magnitude of Cohen’s *d* was rated as follows: 0.20–0.49 “small,” 0.50–0.79 “moderate,” and ≥0.8 as “large,” respectively. Normal distribution of the data was verified by the Kolmogorov–Smirnov test. The threshold for statistical significance was *p* < 0.05.

## Results

All participants tolerated tRNS well, and no side-effects were reported during and after stimulation, with the exception of a slight tingling sensation under the electrodes.

Descriptive statistics of demographics, average stimulation intensity, and baseline MEP characteristics are summarized in Table 1. Age, gender, SI1mV, and baseline MEP did not differ between groups (all values of *p* ≥ 0.104; Table 1).

**Table 1:** Group data for demographic factors, SI1mV (% MSO), and MEP amplitudes of the first dorsal interosseous muscle (mV).

Group	tRNS	*n*	Gender (F/M)	Age (years)	SI1mV	T0 (Baseline)
Congruent	Sham	26	13/13	24.92 ± 4.09	47.69 ± 10.08	1.00 ± 0.28
	Real	26 (1^∗^)	16/10	25.38 ± 3.40	47.58 ± 11.10	0.98 ± 0.27
Mirror-reversed	Sham	26	19/7	24.35 ± 2.97	45.62 ± 10.25	1.01 ± 0.28
	Real	26	14/12	25.92 ± 2.77	48.92 ± 10.62	0.99 ± 0.30
Perceptual sequence	Real	26	18/8	24.12 ± 3.77	44.69 ± 9.40	1.00 ± 0.24
Incongruent (left hand)	Real	26	16/10	23.12 ± 3.88	45.04 ± 11.49	0.97 ± 0.28
Landscape	Real	26	16/10	24.85 ± 4.03	47.08 ± 11.78	0.96 ± 0.20
No execution	Real	26	17/9	23.50 ± 4.04	44.69 ± 10.47	0.99 ± 0.26
Between group		—	*p* = 0.734	*p* = 0.104	*p* = 0.761	*p* = 0.998

*^∗^One female participant was excluded as an outlier in the real tRNS session in Experiment 1. SI1mV = the intensity of TMS required to elicit an average motor evoked potential (MEP) of 1 mV. Baseline MEP, SI1mV, subjects’ age and gender did not significantly differ between experimental groups. Mean values ± Standard deviations are shown for all experimental conditions. Congruent = Experiment 1, Mirror-reversed = Experiment 2, Perceptual sequence = control Experiment 1, Incongruent (left hand) = control Experiment 2, Landscape = control Experiment 3, No execution = control Experiment 4.*

The analysis showed a significant effect of time (*F*_2,200.898_ = 18.13, *p* < 0.0000001) and interaction between time and group (*F*_14,63.663_ = 2.61, *p* = 0.005), but the effect of group was not significant (*F*_7,97.425_ = 1.64, *p* = 0.133).

**FIGURE 2:**
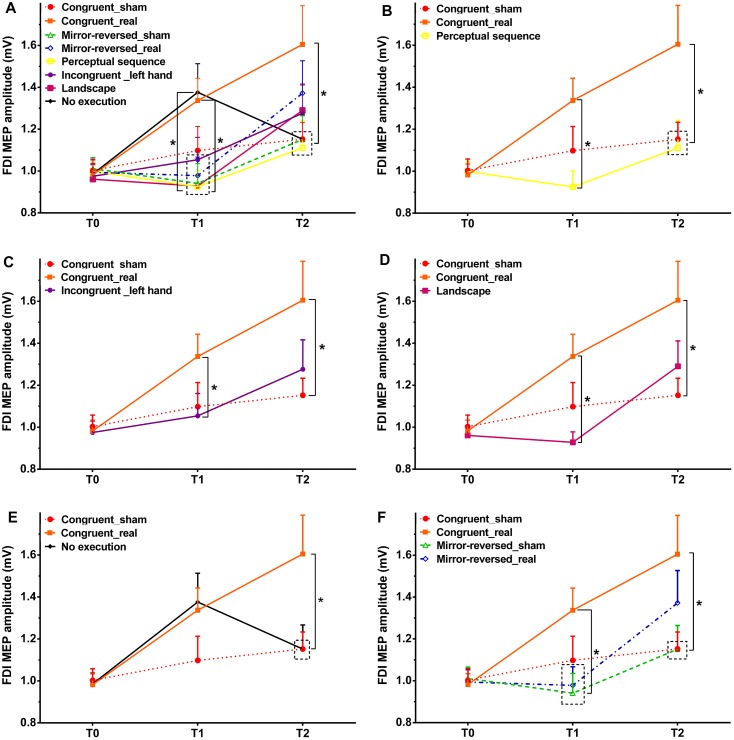
Size of the MEP amplitudes of the FDI muscle before action observation, observing the perceptual sequence or watching a landscape combined with tRNS (T0), as well as immediately after action observation (T1), and after action execution or no execution (T2) in each intervention. Congruent = Experiment 1, Mirror-reversed = Experiment 2, Perceptual sequence = control Experiment 1, Incongruent (left hand) = control Experiment 2, Landscape = control Experiment 3, No execution = control Experiment 4. Sham – sham tRNS, Real – real tRNS. Error bars represent standard error. ^∗^Denotes significant differences between groups (^∗^*p* < 0.05). **(A)** Comparison of MEP amplitudes across all experiments. In order to improve comprehension of **A**, each experiment is presented separately. Comparison of MEP amplitudes between Experiment 1 and control Experiment 1 **(B)**, control Experiment 2 **(C)**, control Experiment 3 **(D)**, as well as control Experiment 4 **(E)**. **(F)** Comparison of MEP amplitudes between Experiment 1 and Experiment 2.

In Experiment 1, as shown by the *post hoc* tests, motor cortical excitability was not significantly different between the sham and real tRNS session at time point T1 (*p* = 0.121, Cohen’s *d* = 0.423), but MEP differed at time point T2 between sham and real tRNS session (*p* = 0.028, Cohen’s *d* = 0.623; Figure 2). Regarding within-group MEP alterations, the results show that MEP amplitudes significantly increased from T0 to T1 (*p* = 0.0004, Cohen’s *d* = 0.833) as well as between T0 and T2 (*p* = 0.001, Cohen’s *d* = 0.900) in the real tRNS session, but did not significantly change between all time points in the sham session as well as between T1 and T2 in the real session.

In Experiment 2, the results showed no significant differences between sham and real tRNS sessions at time point T1 (*p* = 0.675, Cohen’s *d* = 0.079) and T2 (*p* = 0.172, Cohen’s *d* = 0.319). See Figure 2F. MEP amplitudes increased between T0 and T2 (*p* = 0.020, Cohen’s *d* = 0.630) and from T1 to T2 (*p* = 0.008, Cohen’s *d* = 0.610) in the real tRNS sessions. The *post hoc* test showed no significant differences from T0 to T1 in the real session and between all time points in the sham session.

In Experiment 3, the respective *post hoc* test revealed a significant difference at time point T1 between the real tRNS session of Experiment 1 as compared to the perceptual sequence group (control Experiment 1) (*p* = 0.002, Cohen’s *d* = 0.878; Figure 2B), the incongruent group (left hand only, control Experiment 2) (*p* = 0.043, Cohen’s *d* = 0.520; Figure 2C), and the landscape group without movement observation (control Experiment 3) (*p* = 0.001, Cohen’s *d* = 0.969; Figure 2D). For time point T2, the analysis showed a significant difference between the real tRNS group of Experiment 1 as compared to the perceptual sequence group (*p* = 0.029, Cohen’s *d* = 0.605; Figure 2B), and no execution group (control Experiment 4) (*p* = 0.038, Cohen’s *d* = 0.577; Figure 2E). All control experiments did not enhance the MEP amplitudes significantly as compared to the sham condition of Experiment 1 at time point T1 and T2. As compared to the real tRNS session of Experiment 1, MEP amplitudes were smaller in the incongruent group (left hand) and landscape group at T2, but no significant difference were shown between the real tRNS session of Experiment 1 and these two control groups.

## Discussion

The main results of the present study are that tRNS combined with mirror-matched action observation enhanced motor cortex excitability, and that subsequent congruent goal-directed actions enhanced the respective excitability alterations further. In contrast, MEP amplitudes were not significantly enhanced during the time course of the experiment in the sham tRNS session in the congruent experiment and all sessions in control experiments. Furthermore, this effect was not solely based on tRNS, but on the interaction between tRNS and action observation as well as action execution, because without movement execution, the action observation combined with real tRNS-induced MEP amplitudes enhancement vanished after a few minutes. Thus, these findings support our hypotheses that the interaction between tRNS and action observation boosts alterations of motor cortex excitability, and that this effect furthermore enhances execution-related motor cortex excitability. This could be the foundation for a boosting effect of prior action observation combined with tRNS on action execution.

The real tRNS condition of Experiment 1 enhances action observation-related motor cortex excitability to a larger degree, as compared to all control experiments. Post-stimulation MEP amplitudes compared to the baseline value showed a significant increase by prior congruent action observation combined with real tRNS. This implies that tRNS and action observation have synergistic effects on enhancing motor cortex excitability, and that these effects are specific in the sense that they are only observed when the motor cortex involved in action observation is stimulated. For the MEP immediately after action observation, it has been shown in previous studies that action observation alone does not enhance contralateral motor cortex excitability (Sale and Mattingley, 2013). The result of the incongruent experiment is in accordance with previous studies, showing no alteration of ipsilateral motor cortex excitability when TMS-induced MEPs were collected during action observation alone (Sartori et al., 2013, 2014). Previous studies suggest that the ventral PMv plays a pivotal role in mirror neuron discharge in both action observation and action execution (Gallese et al., 1996; Rizzolatti et al., 1996). The PMv includes representations of upper limb movements (Fogassi et al., 2001), including presentations of specific hand-object interactions, and goal-directed movements (Stefan et al., 2005). Thus, at first sight, it is not self-evident why tRNS over M1 should boost observation-related motor cortex excitability. M1 activity, however, is enhanced by PMv activation (Shimazu et al., 2004), and mirror activity in M1 has been demonstrated in humans (Hari et al., 1998). Action observation affects the excitability of connections between PMv and M1 known as action observation pathways (Koch et al., 2010; Lago et al., 2010). Previous research has demonstrated that action observation can enhance the suppression of mu rhythm and mirror neurons activities (Zhang et al., 2018). The population of pyramidal tract neurons in M1 showed a balance of increased and decreased activity during action observation (Hannah et al., 2018). The Network Activity-Dependent Model suggests that stimulation of a node or hub of a specific cortical network spreads to functionally connected brain regions according to effective cortical connectivity (Fertonani and Miniussi, 2017). Thus, tRNS might have enhanced motor cortex excitability via a direct effect on M1 neurons activated by premotor mirror neurons, by indirect activation of respective premotor neurons, or both. An unspecific effect of tRNS independent from action observation-related neuronal activation can be ruled out because tRNS was inefficient in the incongruent experiment, the perceptual sequence group and the landscape group. This implies that tRNS works primarily in conjunction with respective task-related activity affecting M1 activity.

For post-action execution, the results revealed a specific enhancement of motor cortex excitability in the congruent/real tRNS experiment, but no difference between the sham tRNS session in the congruent experiment and all other sessions. This result indicates that combining action execution and prior congruent action observation with real tRNS has synergistic effects on task-related motor cortical excitability. It can be suggested that the task-related neuronal network was more efficiently linked when participants observed the relatively complex movement under tRNS, which then boosted task-related network activity enhancement during performance of the same task. This effect is supra-additive, because execution after congruent observation alone did not significantly enhance excitability, and tRNS without action observation as well as observing a perceptual sequence during tRNS resulted in a smaller excitability enhancement after task execution. Action observation activates the connection between the ventral PMv and M1, and action execution activates connections between the dorsal PMv and M1 or between the supplementary motor area and M1 (Stefan et al., 2008; Cantarero et al., 2011). When the information from these anatomically different routes onto M1 converge through a way of similar neuronal mechanisms (Stefan et al., 2008) they may potentiate specific task-related neuronal activity. For without movement execution, the MEP amplitude enhancement observed after action observation combined with real tRNS vanished after a few minutes. Thus, action execution is required to stabilize the MEP enhancement generated by combined tRNS/action observation, and this might be accomplished via a respective effect of action execution on M1 neurons previously activated by congruent action observation combined with tRNS.

The present study has some limitations. We did not test the effect of action observation combined with tRNS and subsequent action execution on motor performance directly, thus we have no information available if the respective excitability enhancement was associated with improved performance. This should be the topic of consecutive studies. In this study, we used large electrode sizes over M1, which limits specificity of stimulation regarding M1 effects. We furthermore did not aim to develop an optimized stimulation protocol. Both electrodes positioned over brain regions involved in the task under test would have been interesting. This would allow to explore the motor network contribution to task-related excitability changes, and to explore if network stimulation induces superior effects also regarding task-related physiology, as recently shown for the motor cortex at rest (Fischer et al., 2017). We only investigated healthy young subjects, so further studies will need to explore task-related alterations of motor cortex physiology in elderly people or populations of patients.

## Conclusion

Action observation is considered as a therapeutic strategy in the context of motor rehabilitation because it activates motor cortical areas also involved in action execution (Ertelt et al., 2007), and thus might enhance performance. In the current study, we have demonstrated that the interaction between action observation and tRNS can enhance performance-related motor cortical excitability, and that this effect is specific for the combination of task-related interventions. Congruent action observation combined with real tRNS preceding action execution furthermore boosted execution-dependent excitability enhancements. Since task-related cortical excitability alterations are indicative for neuroplastic processes, this might be a hint that combination of action observation with tRNS might be suited to enhance task performance. This might turn out to be an effective neurorehabilitation strategy to enhance physical therapy in future.

## Author Contributions

FQ and MN contributed to the conception, design of the experiment, and revised it critically for important intellectual content. FQ and VZ collected the data. FQ drafted the manuscript. All authors contributed to the analysis and interpretation of the data and approved the final version of the manuscript.

## Conflict of Interest Statement

The authors declare that the research was conducted in the absence of any commercial or financial relationships that could be construed as a potential conflict of interest.
